# Determining biomarkers for evaluation and diagnosis of hereditary angioedema

**DOI:** 10.1002/clt2.12202

**Published:** 2022-10-12

**Authors:** Umesh Singh, Jonathan A. Bernstein

**Affiliations:** ^1^ University of Cincinnati College of Medicine Cincinnati Ohio USA

**Keywords:** biomarkers, differentially expressed genes, HAE attacks, HAE with normal C1 inhibitor, hereditary angioedema, heterogeneity, IL‐1b, beta1/beta3‐integrin, IL‐6, immunology, immunotherapy, kallikrein‐bradykinin, other, PLAUR gene, RNAseq

## Abstract

**Rationale:**

Kallikrein‐bradykinin‐forming cascade is known to cause hereditary angioedema (HAE) acute angioedema (AE) attacks. Further research of HAE attacks is needed to explain disease heterogeneity, predict treatment response and identify biomarkers for monitoring HAE attacks. Differential expression of the microvascular endothelial cell‐surface receptors for example, g‐C1qR, cytokeratin‐1, and plasminogen‐activator‐urokinase‐receptor (PLAUR) were hypothesized as biomarkers of AE attacks.

**Method:**

To understand HAE attacks, the differentially expressed genes (DEGs) in RNAseq and mi‐RNAseq data of total RNA extracted from skin biopsies of lesional versus non‐lesional skin collected during and between attacks in Type‐1 HAE patients (*n* = 11; F:M = 8:3) were compared. To understand the HAE variants, DEGs in skin biopsies from HAE with normal C1 inhibitor (*n* = 5, F:M = 5:0), and non‐HAE (*n* = 7; F:M = 3:4) patients were compared. Gene‐set enrichment analyses and regulator effects analysis of these DEGs identified biological pathways in HAE attacks and their regulators.

**Results:**

PLAUR gene, encoding urokinase‐type plasminogen activator (u‐PAR), was constitutively over‐expressed in HAE‐Type‐1 versus non‐HAE controls suggestive of overactive u‐PAR‐mediated signaling via binding to Factor‐XII. Baseline PLAUR expression was associated with severe AE (*p* = 0.05). The 18 significant DEGs investigated between baseline and AE attack samples in Type1‐HAE were enriched in beta1/beta3‐integrin cell surface interactions and IL‐6‐mediated signaling. Regulator effects analysis suggests a role for IL‐1b in HAE flares. AKT2, the mRNA regulated by the differentially‐expressed miR‐184A, was also associated with HAE attacks.

**Conclusion:**

Angiopoetin‐activated *β*1‐integrin signaling pathways causing endothelial destabilization, and avid binding of factor XII to u‐PAR are possible novel mechanisms for progression of the endothelial kinin‐bradykinin‐forming cascade in HAE attacks.

## 1 BACKGROUND

The kallikrein bradykinin‐forming cascade is recognized as the primary mechanistic pathway for hereditary angioedema (HAE) swelling attacks. Although more effective HAE therapies have evolved over the past 15 years, further research of disease mechanism(s) is needed to explain disease heterogeneity, predict treatment response and identify biomarkers for monitoring disease during and between attacks. Clinically, characterization of HAE swelling attacks is difficult to predict because of their heterogeneity within and between patients with respect to frequency, location, severity and triggers such as psychological and physical stimuli that induce an episode.^1^ Thus, there are current knowledge‐gaps regarding biomarkers that can predict the onset and severity and response to treatment of HAE attacks.

The pathogenesis of HAE involves complex interaction of the complement, contact and fibrinolytic pathways. The primary mutations for HAE Type I and II involve the serine protease inhibitor G1 but more recently a number of genetic factors including factor XII (F12), plasminogen (PLG) and mutations in angiopoietin 1 have been found to result in increased bradykinin 2 receptor mediated signaling leading to increased bradykinin production. Additionally, ANGPT1 gene is known to perturb the cytoskeletal assembly of vascular endothelial cells.^2^

Because of the possible involvement of the endothelial cell microenvironment in the pathogenesis of HAE flares, it was hypothesized that differential expression of the microvascular endothelial cell‐surface receptors such as g‐C1qR, cytokeratin‐1, and plasminogen activator urokinase receptor (PLAUR) could be novel biomarkers for disease activity. These molecules exist in the endothelial cell membrane as the biomolecular complexes, gC1qR‐cytokeratin 1 and cytokeratin 1 – urokinase‐type plasminogen activator (u‐PAR), which primarily bind high molecular weight kininogen‐prekalikrein complex and F12, respectively.^3^

This study aimed to determine the expression profiles of endothelial cell surface receptors, (e.g., plasminogen activation receptor ‐ uPAR) from skin biopsies of patients with HAE Type 1 and HAE with normal C1 inhibitor (HAEnCI) between and during swelling attacks. In addition, investigation of the upstream regulators and molecular networks for such differentially expressed genes (DEGs) was performed to understand mechanisms of acute angioedema attacks based on canonical pathways from existing medical knowledge. Finally, using the regulator effects analysis, we also predicted novel pathways from predicted upstream regulators to downstream molecular/biological functions based on their common association with the DEGs.

## 2 METHODS

### 2.1 Demographics of study subjects

To understand the mechanisms of HAE Type 1 angioedema attacks (referred to as flares in this manuscript), skin biopsies from consented HAE Type 1 subjects (*n* = 11; F:M = 8:3) were collected during flares and during quiescent (asymptomatic) periods that were separated by at least 7 days after completion of the flare. These biopsies were collected from edematous skin at the site of the HAE flare and from an adjacent area of skin after complete resolution of the attack. Skin biopsies during and in between swelling episodes were also obtained from HAE patients with normal complement (*n* = 6, F:M = 5:0). The HAE normal complement subjects all endorsed a family history for angioedema and were tested negative for the F12 mutation. Screening for other known mutations was not performed. A single skin biopsy was obtained from non‐HAE patients (*n* = 7; F:M = 3:4) for comparison to HAE Type I samples.

#### 2.1.1 Inclusion criteria

(1) Ages 18–75 years, (2) confirmed HAE Type 1 (low C1INH), or HAEnCI for the comparison group, (3) patients using on‐demand therapy only, and (4) ability to provide informed consent. Non‐HAE subjects without other allergic disorders were enrolled as the control group.

#### 2.1.2 Exclusion criteria

(1) Pregnancy, (2) cognitive disability, (3) urticaria, (4) acquired angioedema (low C1q), (5) ACE Inhibitor‐induced angioedema, (6) clinical response to H1‐antihistamines, (7) evidence of chronic diseases involving the microvasculature for example, coronary artery disease, diabetes or chronic renal failure not stable per clinical judgment of the investigator, (8) on estrogen oral contraceptives, and (9) prophylactic HAE therapy.

### 2.2 Collection of skin biopsy

See Appendix Methods S1.

### 2.3 Total RNA extraction from skin biopsies

See Appendix Methods S1.

### 2.4 Collection of blood samples and RNA purification

Five ml of whole blood was collected in RNA stabilizing blood tubes and stored at −80°C until further processing (see Appendix Methods S1). Total RNA with miRNA were extracted from the blood sample using the PAXgene blood miRNA procedure as described in the vendor's manual (Qiagen).

### 2.5 RNA‐seq for differential gene expression profiling in skin and blood samples

See Appendix Methods S1.

### 2.6 Data analysis for determining DEGs

The differential gene expression analysis between different sample types was performed using the negative binomial statistical model of read counts as implemented in the *edgeR Bioconductor* package.^4^ The cluster analysis of all genes differentially expressed in individual comparisons is performed using the Bayesian infinite mixture models.^5^ The gene set enrichment analysis (GSEA) was performed using the LRpath methodology^6^ as implemented in the CLEAN package.^7^

The DEGs derived from sequenced data were compared between HAE (either Type 1 or HAEnCI) versus non‐HAE controls and within the HAE patients between samples collected during flares and quiescence. These analytical methods attempted to identify biomarkers that could predict flares and disease progression, and thereby identify potential therapeutic agents to control such events. The DEGs were queried within the Ingenuity knowledge base included in the Ingenuity Pathway Analysis® (IPA, Qiagen) software to perform the core analysis that permits interpretation of the biological relevance of expression changes in the omics data and to determine which metabolic and signaling canonical pathways were enriched in the active flare lesions in HAE Type 1 versus normal healthy skin from non‐HAE controls. The core and subsequent comparison analyses using this application are summarized below. Additionally, the regulator effects analysis, based on the comparison analysis, were also performed that explains the upstream regulators expected to affect the target molecules in the dataset.

### 2.7 Association of PLAUR with severity of HAE angioedema attacks

Differential gene‐expression of PLAUR in skin biopsies from HAE subjects at baseline versus non‐HAE controls were regressed on the severity of angioedema attacks in HAE subjects using generalized linear regression assuming a log‐normal distribution of gene expression values. Severity of attacks were determined clinically by the treating HAE specialist.

### 2.8 Core analysis

Gene expression between comparison groups was explored by IPA® using the expression analysis component of the core analysis to determine the canonical pathways that were enriched in the data. This analysis identified relationships, mechanisms, functions, and pathways relevant to the dataset. Subsequently, the significant upstream regulators affecting the molecules in the dataset were predicted. The biological functions significantly over‐represented were determined. Molecular networks biologically relevant for HAE were determined based on the predicted activation or inhibition of regulatory molecules or biological processes. The *p*‐value of overlap calculated using the right‐tailed Fisher's Exact test with Benjamini‐Hochberg correction for multiple testing, and the *z*‐score, indicative of similar or dissimilar biological signatures between analysis for downstream effects or upstream regulators, were used for determining statistical significance.

### 2.9 Comparison analysis

Comparison analysis was performed on the core analysis results from all the comparator groups to identify trends, similarities and differences between those results. Components of such comparison analyses include comparison of the canonical pathways, and the regulator effects analysis. Thus, the canonical pathways commonly represented in the core analysis between the comparison groups (i.e., the canonical pathways in the core analysis from HAE Type 1 flare vs. control, HAE Type 1 flare vs. HAEnCI, and others) were compared. Thereafter, upstream analysis compared the potential upstream regulators for the DEGs between the different comparison groups. Enriched biological and pathological functions based on these regulators and their target molecules were compared. Regulator effects analysis examined connections between upstream regulators, the dataset molecules and downstream functions in HAE pathogenesis, and determined if any specific therapeutic molecules could hypothetically minimize or prevent the differential gene expression thus preventing the pathogenesis of HAE flares.

### 2.10 miRNA analysis from blood samples

The list of differentially expressed miRNA were uploaded to the IPA. Core analyses for each comparison group were performed. Highly restrained stringent filters were used for querying only those molecules and relationships very relevant to humans. Pathway tools were used to generate and test additional hypothesis. The “build” tool was used to determine which canonical or well‐established pathways were associated with the molecules in the study data. Experimentally validated mRNAs, regulated by the differentially expressed miRNA in the data were determined. The canonical pathways, associated with the differentially expressed miRNA experimentally validated to regulate mRNA expression in TargetScan, miRecords, Ingenuity Expert Findings, were investigated. IPA tools were used to customize the networks and pathways that permitted generation of additional hypotheses from the networks identified by these tools.

## 3 RESULTS

### 3.1 Core analysis

#### 3.1.1 HAE Type 1 baseline and flare skin biopsy samples versus control samples

The PLAUR gene that encodes u‐PAR was overexpressed at baseline and its expression further increased during flares in HAE‐Type‐1 patients compared to non‐HAE and HAEnCI controls suggestive of overactive u‐PAR‐mediated signaling via binding to Factor‐XII.^8^ Increased baseline PLAUR expression in HAE Type I patients compared with non‐HAE controls was associated with severe angioedema attacks (*p* = 0.0003), (Appendix Table 1). Using GSEA of the DEGs between HAE Type I baseline and flare samples, 18 DEGs were determined to be enriched in beta1/beta3‐integrin cell surface interactions and IL‐6‐mediated signaling events.

### 3.2 Canonical pathways in core analysis

Appendix Tables 2 and 3 summarize the predicted canonical pathways using core analysis which were necessary for performing the comparison analysis.

### 3.3 Comparison analysis

#### 3.3.1 Comparing canonical pathways

**FIGURE 1:**
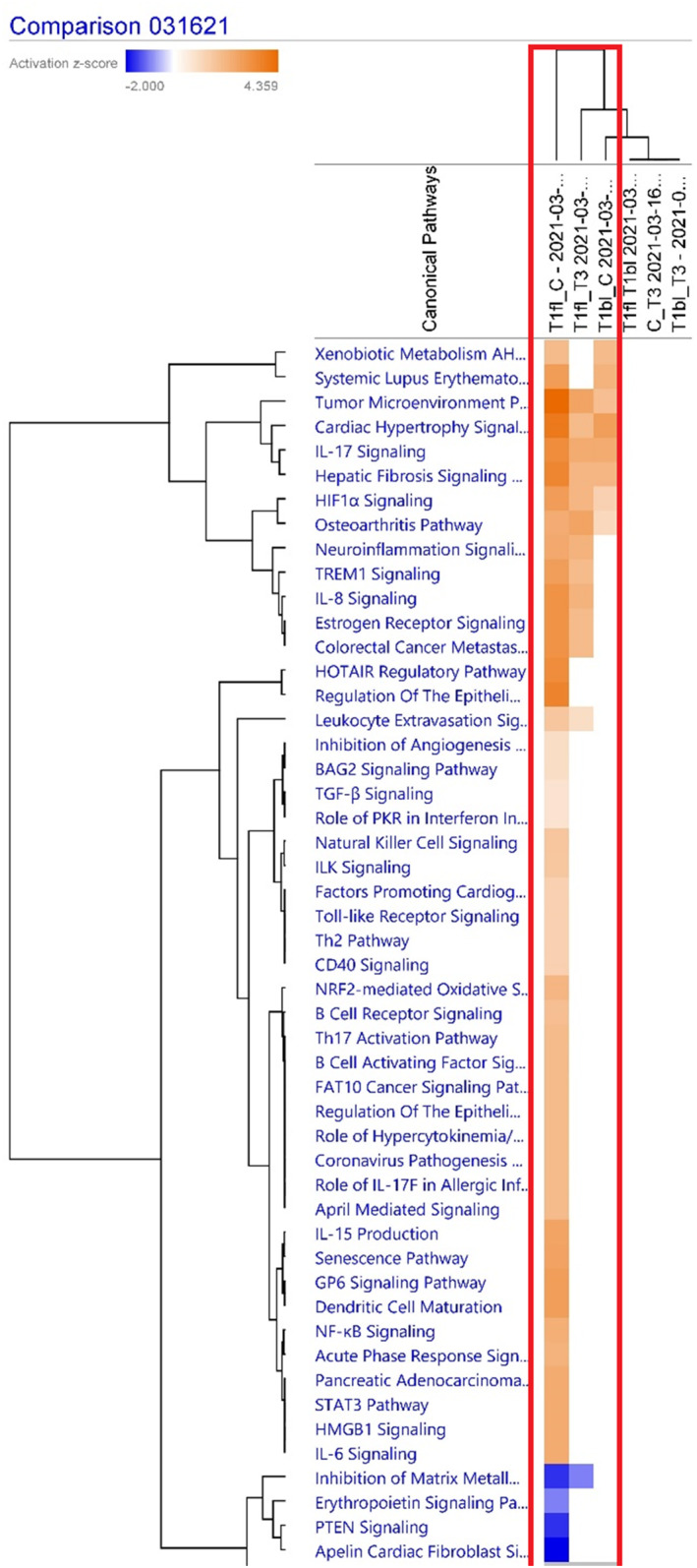
Canonical Pathways between comparison groups in the comparison analysis of skin biopsies—blue represents significant under expression and orange over expression (comparison of interest in the red box were T1fl_C i.e., HAE [Type 1 flare vs. non‐HAE control]; T1fl_T3 i.e., HAE [Type 1 flare vs. HAE with normal C1INH during a flare]; T1bl_C i.e., HAE [Type I HAE baseline vs. non‐HAE controls]). HAE, hereditary angioedema

Figure 1 compares the significant canonical pathways between HAE Type 1 flares versus controls, HAE Type 1 flares versus HAEnCI, HAE Type 1 baseline versus controls, HAE Type 1 flares versus controls versus HAEnCI, and HAE Type 1 quiescence versus HAE nl. The hierarchical clustering method used in this analysis clustered the comparison groups with similar scores. As expected, the HAE Type 1 flare versus controls and HAE Type 1 flare versus HAEnCI samples are grouped towards the top whereas the Type 1 HAE quiescence samples towards the bottom.

Canonical pathways most relevant for the pathogenesis of Type 1 flares were IL17 signaling, neuroinflammation signaling, triggering receptor expressed on myeloid cells‐1 (TREM1) signaling, IL8 signaling, regulation of epithelial mesenchymal transition by Growth Factors Pathway and leukocyte extravasation signaling. Additionally, pathways exclusively upregulated in Type 1 flares versus controls are NK cell signaling, integrin linked kinase signaling, NRF2‐mediated oxidative stress response, Th17 activation pathway, IL15 production and the coronavirus pathogenesis pathway.

Pathways exclusively downregulated in this comparison group were inhibition of matrix metalloproteinase, phosphatase and tensin homolog (PTEN) signaling, erythropoietin signaling pathway.

#### 3.3.2 Comparison analysis of the upstream regulators

**FIGURE 2:**
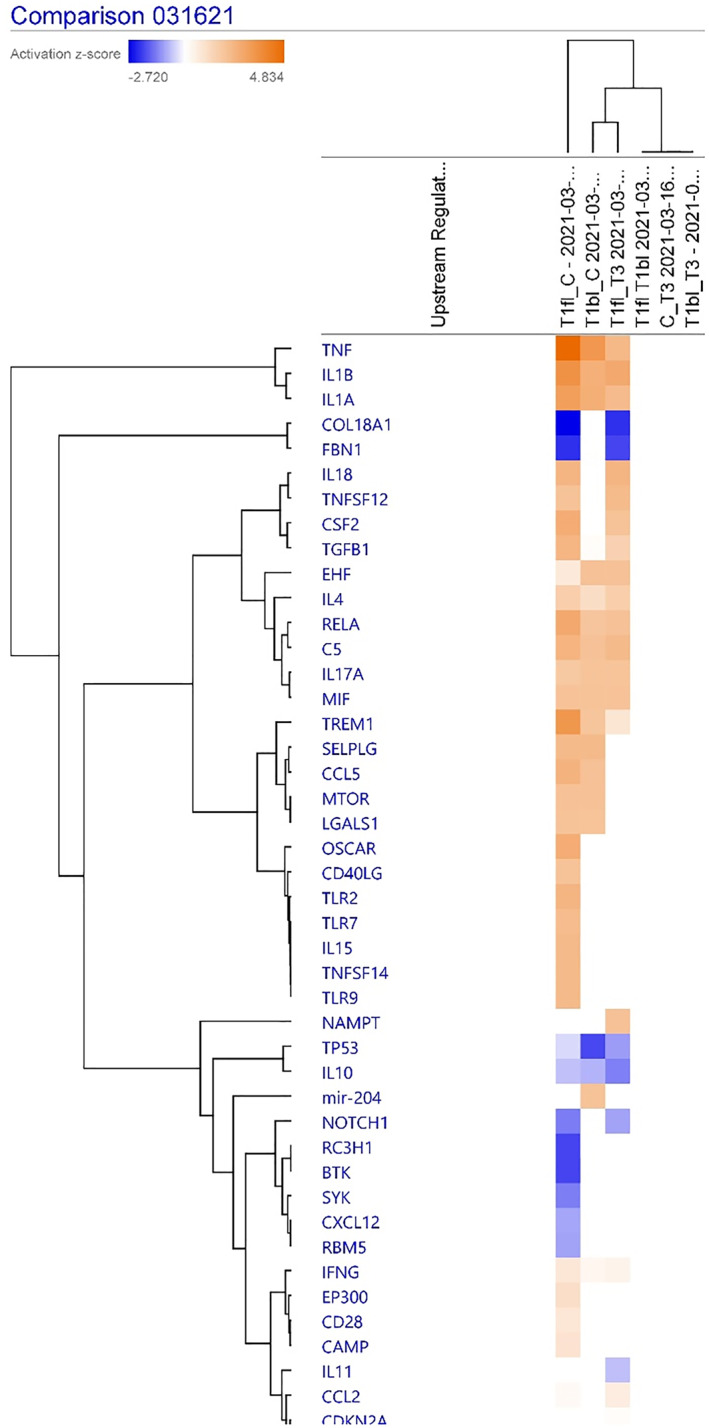
Upstream regulators between comparison groups in the comparison analysis in skin biopsies. – blue represents significant under expression and orange overexpression (comparison of interest were T1fl_C [HAE Type 1 flare vs. non‐HAE control]; T1fl_T3 [HAE Type 1 flare vs. HAE with normal C1INH during a flare]; T1bl_C [HAE Type I HAE baseline vs. non‐HAE controls]). HAE, hereditary angioedema

Figure 2 shows that the upstream regulators significantly predicted to be activated between HAE Type 1 flares versus controls and HAE Type 1 flares versus HAEnCI were TNF, IL1*β*, IL1A, IL18, TNFSF12, CSF2, TGF*β*1, IL4, C5, IL17α, MIF, and TREM1; regulators predicted to be inhibited in these samples were COL18A1, FBN1, TP53, and IL10. In addition, TNF, IL1*β*, IL1A were also predicted to be activated in HAE Type 1 baseline versus control samples implying a baseline activation of these regulators in HAE Type 1 even during quiescence.

#### 3.3.3 Regulator effects analysis

**FIGURE 3:**
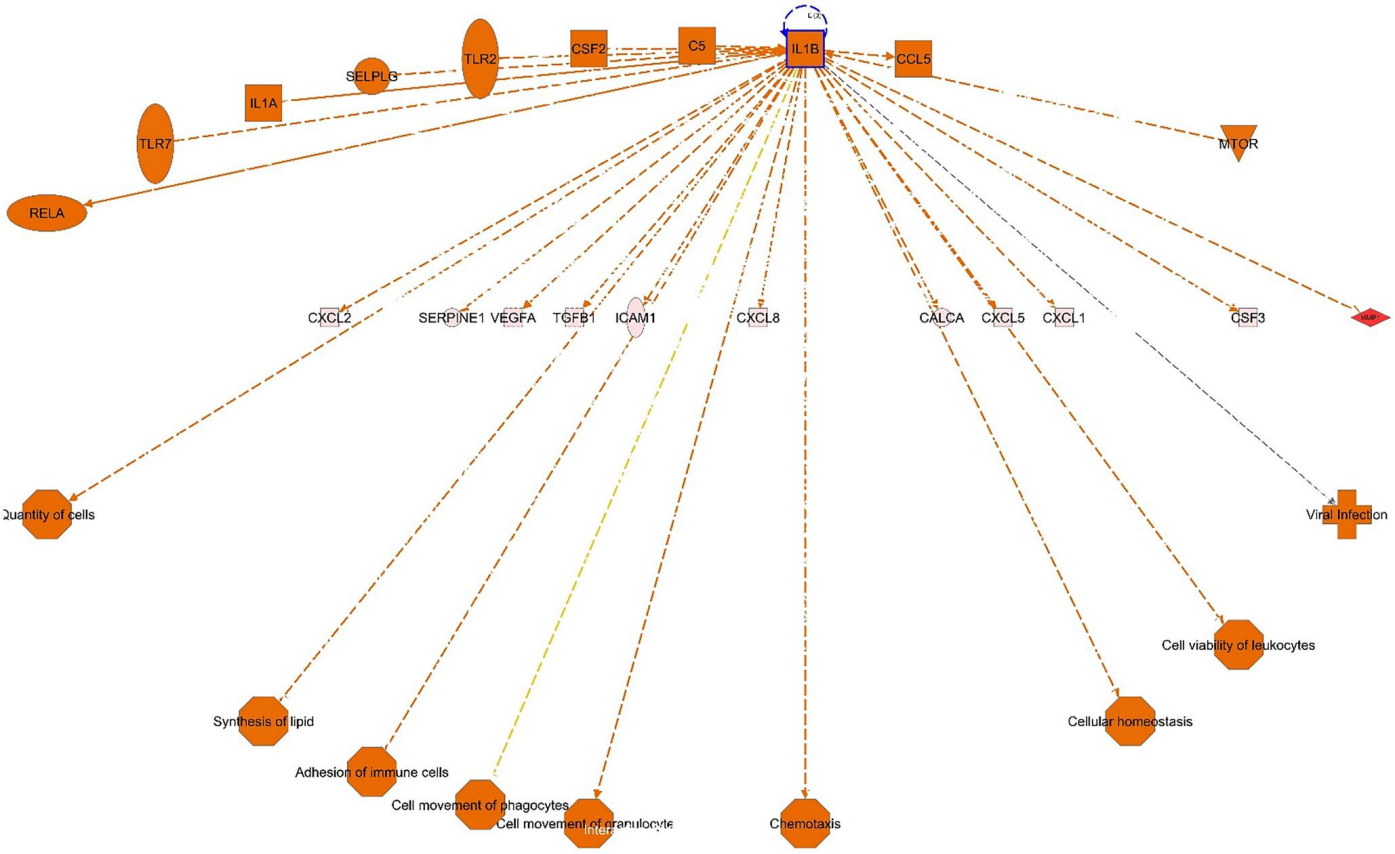
Regulator effects analysis of skin biopsies. IL1‐*β* as an upstream regulator for VEGFA has direct links to several downstream biological functions. Additional novel mechanisms can be hypothesized through indirect links mediated by differentially expressed genes (DEGs) (e.g., VEGFA, CXCL2/5) that can link these regulators with previously unknown or unidentified downstream effects

Table 1 and Figure 3 show the upstream regulators that are predicted to be activated or inhibited based on DEGs in the comparison groups. These regulators may increase or decrease the functional outcomes in the pathogenesis of HAE. Regulator effects analysis generate directional networks based on the study sample DEGs and the existing scientific knowledge allowing for the generation of novel hypotheses. The causal hypotheses are visualized as networks generated by automatically merging the upstream analysis to the diseases and function results with the dataset molecules acting as intermediaries that carry signals from the upstream regulators to the downstream outcomes. The resulting regulator effects networks identifies the potential drug targets and mechanisms of their efficacy.

**TABLE 1:** Regulator effects analysis based on consistency scores

Analysis	Consistency score	Regulators	Target molecules in study samples	Diseases & functions	Known relationship^a^
T1 flare/C	41.29	C5, CCL5, CSF2, IL1A, IL1B, MTOR, OSCAR, RELA, SELPLG, TLR2, TLR7, TLR9, TNFSF14	ADGRE5, CALCA, CCR4, CCR7, CD83, CSF3, CXCL1, CXCL2, CXCL5, CXCL8, CXCR4, CYP27B1, FN1, ICAM1, ISG15, ITGAX, mir‐132, MMP1, MMP9, PLAUR, SERPINE1, TGFB1, VEGFA	Adhesion of immune cells, cell movement of endothelial cells, cell movement of granulocytes, cell movement of phagocytes, cell viability of leukocytes, cellular homeostasis, chemotaxis, differentiation of myeloid leukocytes, interaction of mononuclear leukocytes, lymphocyte migration, quantity of cells, synthesis of lipid, viral infection	36% (61/169)
T1 flare/C	34.18	C5, COL18A1, IL1A, IL1B, MTOR, OSCAR, RELA, TNFSF14	CALCA, CCR4, CSF3, CXCL1, CXCL2, CXCL5, CXCL8, CXCR4, FN1, HGF, ICAM1, MMP1, MMP9, SERPINE1, TGFB1, THBS1, TLR2, VEGFA	Cell movement of granulocytes, cell movement of mononuclear leukocytes, cell movement of phagocytes, cell viability of leukocytes, cellular homeostasis, chemotaxis, differentiation of myeloid leukocytes	29% (16/56)
T1 flare/C	33.47	C5, IL1A, IL1B, MTOR, OSCAR, RELA, SELPLG, TGFB1, TLR7, TLR9, TNF	CALCA, CCR7, CD83, CSF3, CXCL1, CXCL2, CXCL5, CXCL8, CXCR4, FN1, ICAM1, ISG15, MMP9, PLAUR, SERPINE1, SPHK1, TLR2, VEGFA	Cell movement of neutrophils, cell viability of leukocytes, chemotaxis of granulocytes, chemotaxis of phagocytes, quantity of leukocytes, synthesis of reactive oxygen species	35% (23/66)
T1bl/C	20.13	CCL5, IL1B, MTOR, SELPLG, TNF	CXCL1, CXCL2, CXCL8, PLAUR, SERPINE1	Binding of neutrophils, cell movement of granulocytes, chemotaxis of myeloid cells	67% (10/15)
T1 flare/T3	2	IL18	CXCL8, ICAM1, IL6, TIMP1	Cell survival	0% (0/1)
T1bl/C	−4.08	TNF	CXCL1, CXCL2, CXCL8, IL1B, PLAUR, SERPINE1	Chemotaxis of phagocytes	100% (1/1)
T1 flare/T3	−4.92	IL1B	CXCL1, CXCL8, ICAM1, IL6, SERPINE1	Cell movement of myeloid cells	100% (1/1)
T1 flare/T3	−6	C5	CXCL8, ICAM1, IL6, SERPINE1	Cell movement of myeloid cells	100% (1/1)
T1 flare/T3	−6	IL1A	CXCL1, CXCL8, ICAM1, IL6	Cell movement of myeloid cells	0% (0/1)
T1 flare/C	−6	COL18A1	FN1, HGF, ICAM1, THBS1	Binding of mononuclear leukocytes	0% (0/1)
T1 flare/C	−6	IL1B	CXCL1, CXCL8, TGFB1, VEGFA	Tubulation of endothelial cells	0% (0/1)
T1 flare/C	−8.17	TREM1	CCR7, CXCL1, CXCL2, CXCL5, CXCL8, CXCR4	Chemotaxis of myeloid cells, chemotaxis of phagocytes	0% (0/2)
T1 flare/T3	−12.08	TNF	CXCL8, HMOX1, ICAM1, IL6, TIMP1	Cell viability	100% (1/1)

Abbreviations: C, control; T1, HAE Type 1; T3, HAE with normal C1INH.

^a^ Known regulator‐disease/function relationship.

The top networks in this analysis determine the activated upstream regulators relevant in the pathogenesis of HAE flares. The statistically significant networks, shown in Table 1, are ranked based on consistency scores; the highly connected networks with consistent relationships are scored higher (i.e., observed or predicted directions of activity among regulators, dataset molecules and the disease/function are consistent with the expected direction). The “*Known Relationship*” column indicates the percentage of relationship between upstream regulators and biological functions that have been previously reported in the literature. For the first network, 36% of the relationships between upstream regulators and the pathogenesis of acute HAE flares have been reported previously but the remaining relationships have not. Upstream regulators without the relationship lines connecting to a downstream function(s) imply novel associations. This is applicable for multiple pathways that are directed from the upstream regulators to the dataset molecules and the downstream functions. For example, there may be a novel relationship between IL1*β* (the target for canakinumab/colchicine) and differentiation of myeloid leukocytes, interaction of mononuclear leucocytes, lymphocyte migration and movement of endothelial cells (Figure 3). However, there is an established relationship between IL1*β* and several dataset molecules such as VEGFA, CXCL8 and TGF*β*1.^9^, ^10^, ^11^, ^12^ In this study, IL1is shown to be significantly overexpressed in patients with HAE Type 1 versus non‐HAE controls. As mentioned in Table 1, it is one of the upstream regulators for the interconnected pathways leading to acute angioedema flares. This finding is supported by previous studies that have shown IL1 to stimulate B‐1 bradykinin receptors, and to stimulate endothelial cells to secrete urokinase that can bind to u‐PAR and stimulate fibrinolysis.^13^, ^14^ In addition there are other known relationships between these DEGs in the dataset and the mechanism of Type 1 HAE flares. Thus, from the regulator effects analysis it can be hypothesized that IL1*β* promotes HAE flares.

##### Baseline versus control sample miRNA analysis

In the list of differentially expressed miRNA between baseline HAE samples versus non‐HAE controls there were 11 miRNAs (let‐7a‐3p, mir‐127‐5p, miR‐135a‐1‐3p, miR‐184, miR‐200b‐3p, miR‐3605‐3p, miR‐409‐5p, miR‐412‐5p, miR‐4654, miR‐4669, miR‐6716‐3p) that were highly predicted to target 181 mRNAs, from which two miRNAs (miR‐200b‐3p and miR‐184) with expression fold‐change of 3.9 and 3.2, respectively, had been experimentally verified to target 6 mRNAs: MARCkS, AKT2, PLCG1, NFATC2, ELMO2, and PTEN (source: TargetScan, miRecords, Ingenuity Expert Findings). The enriched pathways associated with the corresponding mRNA relevant to the understanding of HAE, include phagosome formation, phospholipase C signaling, synaptogenesis signaling pathway (MARCKS), acute phase response, interleukin signaling (AKT2), CCR5 signaling (PLCG1), B‐cell activating factor signaling, B‐cell receptor signaling (NFATC2), CXCR4 signaling, phagosome formation (ELMO2), B‐cell receptor signaling, Fc‐gamma receptor‐mediated phagocytosis in macrophages/monocytes and ICOS‐ICOSL signaling in T‐helper cells. Signaling pathways such as IL‐7, IL‐9, IL‐15, signaling in lymphocytes, tight‐junction signaling, TLR signaling, and cellular immune response were specifically selected to filter these miRNAs and the targeted mRNA.

##### Differentially expressed miRNA between HAE baseline versus flare blood samples

miR‐1‐3p and miR‐184, with expression fold‐changes of 6.7 and 4.4 respectively, were two differentially expressed miRNAs between the blood samples collected during HAE Type I quiescence and flares. Together these miRNAs target 53 experimentally validated mRNAs that are enriched in several canonical pathways including IL‐15 production, IL‐17 signaling, VEGF‐signaling, IGF‐1 signaling, IL‐8 signaling, G‐protein coupled receptor signaling, LPS/IL‐1 mediated inhibition of RXR function, IL‐4 signaling, and Th17 activation pathway (Figure 5). ATK2 was one of the most significant mRNAs enriched in these canonical pathways. The most relevant molecules associated with AKT2 were TNF, IL‐4, IL‐15, TGF‐beta, and CSF2 as determined by the Ingenuity knowledge base; the associated biological functions were decreased production of albumin, leucocyte infiltration, activation of immune cells, adhesion of immune cells, and cellular movement of lymphocytes.

## 4 DISCUSSION

Genomic analysis of skin biopsies from HAE‐Type‐1 versus Control/HAEnCI suggest novel mechanistic pathways in HAE attacks, including angiopoietin‐activated *β*1‐integrin signaling leading to endothelial destabilization, and avid binding of F12 to u‐PAR supporting a model for progression of the kinin‐forming cascade on endothelial cells.^15^

Basal levels of reactive oxygen species and oxidative proteins have previously been determined to be significantly higher in HAE versus controls, signifying abnormalities in redox homeostasis caused by an imbalance between the production of reactive oxygen and the detoxification of reactive intermediates.^16^, ^17^ These reports from previous studies support the results of the canonical pathways identified in this study as NRF2‐mediated Oxidative Stress Response pathways, in Figure 1 from the comparison analysis, is upregulated in biopsy samples from HAE Type 1 flares versus controls. Other triggers such as common bacterial and viral infections, have also been known to trigger angioedema attacks in HAE,^18^, ^19^, ^20^ which is supported by upregulation of IL17 signaling and *Coronavirus Pathogenesis pathways* in the comparison analysis. IL17 signaling was upregulated in both flare and baseline samples. The latter finding is supported by previous studies that have demonstrated significantly higher IL‐17 levels in HAE subjects compared to healthy controls. Th17 cells are known to produce IL‐17A, IL‐17F, IL‐22 and IL‐21 cytokines that clear extracellular microbes in the gastrointestinal tract, lungs, and skin.^18^ Accordingly, IL‐17Α and IL‐17F are key cytokines for the recruitment, activation, and migration of neutrophils and can induce pro‐inflammatory mediators such as IL‐6, TNF‐*β*, IL‐1, GM‐CSF and chemokines in fibroblasts, endothelial cells, airway smooth muscle cells, and epithelial cells. These concepts align with the significant upstream regulators for the canonical pathways, such as TNF, IL‐1*β*, IL‐1α, IL‐17α, TGF‐*β*, that are predicted to be activated (Figure 2) in HAE Type 1 flares and baseline samples versus controls or HAEnCI patients.

Among the pathways predicted to be inhibited are PTEN signaling and inhibition of matrix metalloproteinases. These pathways are known to be responsible for maintaining tissue homeostasis.^21^, ^22^ Thus, dysregulation of tissue homeostasis may be an additional factor for triggering angioedema flares.

Through the regulator effects analysis, this study determined the hypothetical role of IL1‐*β* in the development of acute flares. Figure 3 shows direct links between IL1‐*β* (the regulator for the VEGFA) and cell movement of mononuclear leucocytes but not between other downstream biological functions. However, there are links between VEGFA and cellular homeostasis and differentiation of myeloid leukocytes. Thus, there may be novel pathways mediated by IL1‐*β* via VEGFA not reported previously to be involved in the HAE pathogenesis. Drug targets for such upstream regulators could be effective as novel molecules for treating or preventing acute angioedema episodes in HAE Type 1. Thus, the regulator effects analysis as described in Figure 2 and Table 1, can be investigated further to determine several other novel pathways mediated by other regulators that can be controlled by known and novel drug targets.

**FIGURE 4:**
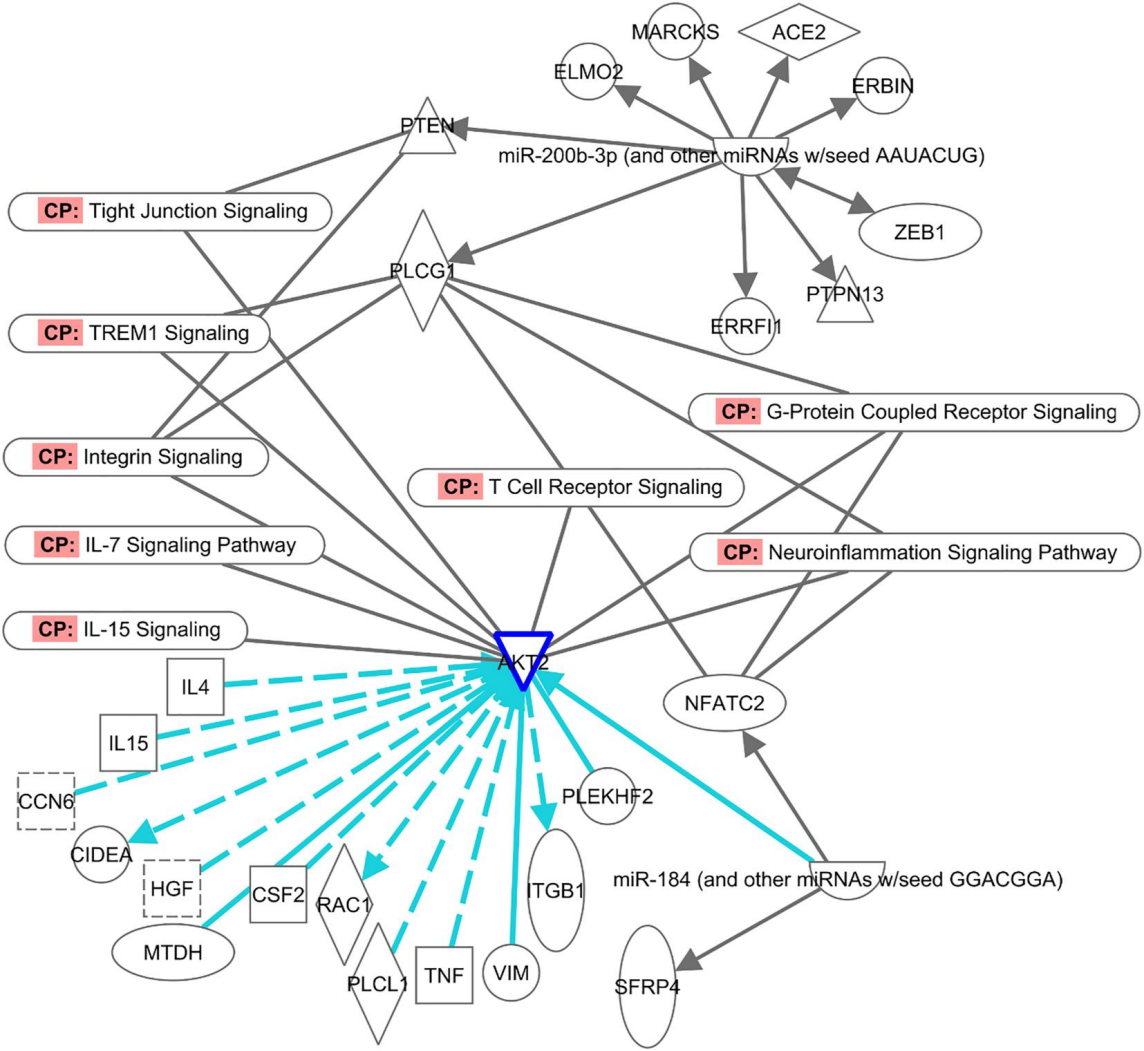
Network analysis of differentially expressed miRNA between HAE baseline versus non‐HAE control blood samples. Canonical pathways associated with mRNAs regulated by differentially expressed miRNA (i.e., miR‐184 and miR‐200b‐3p) are shown. “Build” tools in IPA were used to determine the molecules associated with AKT2 (mRNA regulated by miR‐184). HAE, hereditary angioedema

**FIGURE 5:**
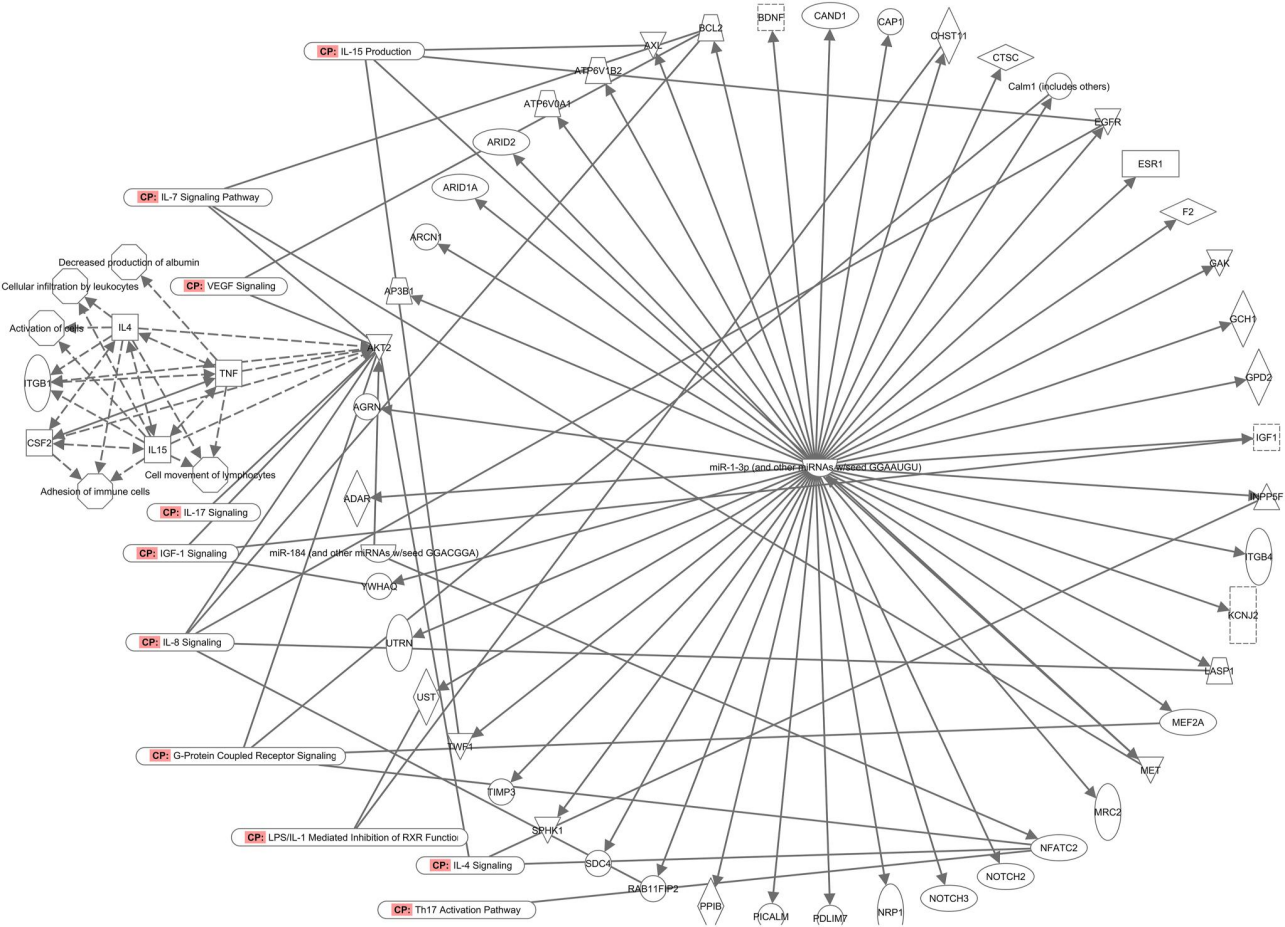
Network analysis of differentially expressed miRNA between HAE baseline versus HAE Type I flare blood samples. Canonical pathways associated with mRNAs regulated by differentially expressed miRNA (i.e., miR‐184 and miR‐1‐3p) are shown. “Build” tools in IPA were used to determine the molecules associated with AKT2 (mRNA regulated by miR‐184). HAE, hereditary angioedema

Analysis of the differentially expressed miRNAs between baseline (i.e., quiescence) and control samples identifies AKT2, directly regulated by miRNA‐184, as one of the key mRNAs that might be involved in the pathogenesis of HAE (Figure 4). The miR‐184 is also determined to be differentially expressed in flare (vs. baseline) samples. Thus, miRNA‐184 can be regarded as a significant biomarker in HAE pathogenesis. Expression of AKT2, one of the mRNAs regulated by miR‐184, may be an important factor in the development of HAE symptoms through its association with IL‐4, IL‐15, TNF, CSF2, and TGF‐beta that mediate several signaling pathways such as IL‐5 production, IL‐4, IL‐8 and IL‐17 signaling, VEGF signaling and others as shown in Figure 5.

Strengths of this study are that it obtained skin and biopsy samples during and in between HAE flares and obtained cohort control groups for comparison. In addition, the identification of previously reported upregulated pathways validates the results of this study while also identifying other potential biomarkers and biologic pathways involved in the pathogenesis of HAE. One limitation of this study is that samples collected in this study were limited to HAE patients attending only one clinic and were thus representative of the patient population from a specific geographical and demographic segment. Thus, the study samples may not fully represent the heterogeneity of the entire HAE population. However, the results based on these samples may be considered reasonable approximations of the desired precision of estimates for HAE patients.

Another potential limitation of this study could be the size of the groups analyzed. However, our initial power assumptions predicted statistically significant differences would be found between HAE Type 1 patients during a flare compared to baseline levels and controls. Interestingly, HAEnCI subjects did not exhibit any of the up or downregulated pathways identified for HAE Type 1 patients and actually more closely resembled the non‐HAE control population suggesting other unique biologic pathways are involved in the form or angioedema. Additionally, the study results were not tested for their generalizability to a broader population. However, our findings of significant changes in biologic pathways in skin biopsies of HAE Type 1 patients during a flare reported by other centers suggests otherwise.

In conclusion, this study investigated the most relevant biologic pathways highly associated with the pathogenesis of Type I HAE flares. It is the first study to compare the pathogenesis of HAE Type 1 versus HAEnCI subjects. Several biomarkers and novel pathways were identified as possible mechanisms for HAE Type 1 flares. These results add more evidence to the roles of endothelial dysfunction, mediated by TREM, IL8, and IL17 signaling pathway, and matrix metalloproteinases, mediated by downregulation of COL18A1 and FBN1, in differentiating HAE Type 1 flares versus HAEnCI patients. As hypothesized, overexpression of the PLAUR gene that encodes u‐PAR was one of the most pertinent mechanistic pathways upregulated during an HAE Type 1 flare. Among the canonical pathways, imbalance in the handling of oxidative stresses mediated by diverse triggers, interleukin signaling as a response towards microbial/viral infections, and downregulation in the synthesis of intercellular adhesion molecules were also very relevant. From Figure 2, it is possible to speculate that IL1‐beta, COL18A1 and FBN1 expression patterns that have high z scores at baseline could predict the prodromes leading to a flare in HAE type 1 patients. Furthermore, AKT2, one of the mRNAs regulated by miR‐184A, was highly associated with an HAE flare, and therefore could be a potential biomarker to confirm subtle angioedema episodes especially those involving the abdomen which can sometimes be difficult to differentiate from other causes of abdominal pain. Finally, additional analysis of regulator effects in skin biopsy and peripheral blood samples identified in this study revealed novel pathways that may be potential upstream drug targets for managing acute flares.

## AUTHOR CONTRIBUTIONS

**Umesh Singh**: Conceptualization (Equal); Data curation (Lead); Formal analysis (Lead); Methodology (Equal); Project administration (Equal); Software (Lead); Validation (Equal); Visualization (Lead); Writing – original draft (Equal); Writing – review & editing (Equal). **Jonathan A. Bernstein**: Conceptualization (Equal); Funding acquisition (Lead); Investigation (Lead); Methodology (Equal); Project administration (Equal); Resources (Lead); Supervision (Lead); Validation (Equal); Writing – original draft (Equal); Writing – review & editing (Equal).

## CONFLICTS OF INTEREST

Umesh Singh: Nothing to disclose. Jonathan A. Bernstein: PI, consultant, speaker: Takeda/Shire, CSL Behring, Pharming, Biocyrst; PI, consultant: Biomarin, Kalvista, Ionis; Consultant:; Pharvaris, Astria.

## Supporting information

Supplementary Material S1

Supplementary Material S2
