# Supplementary material for: Determining biomarkers for evaluation and diagnosis of hereditary angioedema
Source: Clin Transl Allergy. 2022 Oct 12;12(10):e12202. doi: 10.1002/clt2.12202 (PMC9557132; doi:10.1002/clt2.12202)
Supplement: Supplementary file 1 — Supplementary Material S1 [file CLT2-12-e12202-s002.docx]

**APPENDIX METHODS**

1. **METHODS**
   1. **Collection of Skin Biopsy**

The biopsy site was cleaned with isopropyl alcohol and anesthetized using lidocaine. Using a punch biopsy tool, a 4-mm diameter of the skin was biopsied and preserved in RNA-stabilizing media (*RNAlater*, ThermoFisher) until total RNA extraction.

- 1. **Total RNA Extraction from Skin Biopsies**

PureLink® RNA Mini Kit (ThermoFisher) was used to extract total RNA from the skin biopsies. Biopsy samples were lysed and homogenized in the presence of guanidinium isothiocyanate. Thereafter, ethanol was added to the sample and then processed through a spin cartridge with silica-based membrane that binds RNA. Impurities were removed by subsequent washing. The purified total RNA eluted in RNase-free water were used for directional RNA-sequencing.

- 1. **Collection of Blood Samples and RNA purification**

Five mL of whole blood was collected in RNA stabilizing blood tubes and stored at -80℃ until further processing. Total RNA with miRNA were extracted from the blood sample using the PAXgene blood miRNA procedure as described in the vendor’s manual (Qiagen).^3^

- 1. **RNA-Seq for Differential Gene Expression Profiling in Skin and Blood Samples**

The RNA quality was measured by Bioanalyzer (Agilent, Santa Clara, CA). Using 1 μg total RNA as input, globin mRNA and rRNA depleted RNA was isolated by using the Globin-Zero Gold kit (Illumina, San Diego, CA). Next, the NEBNext Ultra II Directional RNA Library Prep Kit (New England BioLabs) was used for library preparation according to the manufacturer’s instruction, and sequenced via NextSeq 550 (Illumina, San Diego, CA) under the setting of single read 1×85 bp to generate ~50 million pass filter reads per sample.
