# Supplementary material for: Determining biomarkers for evaluation and diagnosis of hereditary angioedema
Source: Clin Transl Allergy. 2022 Oct 12;12(10):e12202. doi: 10.1002/clt2.12202 (PMC9557132; doi:10.1002/clt2.12202)
Supplement: Supplementary file 2 — Supplementary Material S2 [file CLT2-12-e12202-s001.docx]

**APPENDIX TABLES**

| **Appendix Table 1**. Association of PLAUR expression with the severity of acute angioedema attacks in HAE Type 1 | | | |
| --- | --- | --- | --- |
| **HAE Angioedema Attack Grade** | **Comparison Group** | **Average difference (±95% CL) in log2-fold gene-expression** | **Adjusted p-value** |
| Mild - Moderate | Control | 2.5 (0.5, 11.0) | 0.26 |
| Severe | Control | 44.5 (8.6, 230.3) | 0.0003 |

| **Appendix Table 2**. Canonical Pathways of Comparison Between ***HAE*** ***Acute Angioedema Attack vs. Non-HAE Control Skin Biopsy Samples*** | | | | |
| --- | --- | --- | --- | --- |
| **Canonical Pathways** | **-log(p-value)** | **Ratio** | **z-score** | **Molecules** |
| Cardiac Hypertrophy Signaling (Enhanced) | 1.81 | 0.0329 | 3.873 | CXCL8,FGFR1,IL18R1,IL1RL1,IL7R,ITGA5,MAP2K3,NFATC1,NFATC4,NFKB2,PDE3A,RCAN1,  TGFB1, TGFB3, WNT9A |
| Hepatic Fibrosis Signaling Pathway | 7.19 | 0.0657 | 3.545 | COL11A2,COL3A1,CXCL8,FGFR1,ICAM1,IL1RL1,IRAK2,ITGA5,MAP2K3,MMP1,NFKB2,PDGFRA,  PDGFRB, PGF, SERPINE1, SMAD7,SNAI1,SOD2,TGFB1,TGFB3,TIMP1,VEGFA,WNT9A |
| HOTAIR Regulatory Pathway | 4.17 | 0.0738 | 3.317 | ICAM1,MMP1,MMP10,MMP19,MMP3,MMP9,NFKB2,TGFB1,TWIST1,TWIST2,WNT9A |
| Colorectal Cancer Metastasis Signaling | 3.46 | 0.0542 | 3.162 | LRP1,MMP1,MMP10,MMP19,MMP3,MMP9,NFKB2,PGF,TGFB1,TGFB3,TLR2,VEGFA,WNT9A |
| IL-8 Signaling | 2.68 | 0.0524 | 3.162 | CXCL1, CXCL8,HBEGF,HMOX1,ICAM1,IRAK2,ITGAX,MMP9,PGF,VEGFA |
| Estrogen Receptor Signaling | 1.34 | 0.0326 | 3.162 | MMP1, MMP10, MMP19, MMP3, MMP9, NFKB2, PGF, SNAI1, SOD2, VEGFA |
| Systemic Lupus Erythematosus In B Cell Signaling Pathway | 2.74 | 0.0474 | 2.887 | CD79B, CXCL8, ISG15, ISG20, NFATC1, NFATC4,NFKB2,RASGRP2,SYNJ2,TGFB1,TGFB3,TRAF1 |
| TREM1 Signaling | 5.66 | 0.136 | 2.828 | CD83,CXCL2,CXCL8,ICAM1,IL1RL1,ITGA5,ITGAX,NFKB2,TLR2 |
| GP6 Signaling Pathway | 3.06 | 0.0708 | 2.828 | COL11A2,COL23A1,COL3A1,COL4A1,COL4A2,COL6A2,COL6A3,RASGRP2 |
| Dendritic Cell Maturation | 2.06 | 0.0488 | 2.828 | CCR7,CD83,COL11A2,COL3A1,ICAM1,NFKB2,RELB,TLR2 |
| Senescence Pathway | 3.61 | 0.0532 | 2.673 | CXCL8,GADD45A,MAP2K3,NFATC1,NFATC4,NFKB2,SERPINE1,SMAD6,SMAD7,SOD2,SQSTM1,TGFB1, TGFB3,TLR2 |
| IL-15 Production | 2.35 | 0.0603 | 2.646 | DYRK3,EPHB2,FGFR1,MAP2K3,NFKB2,PDGFRA,PDGFRB |
| Neuroinflammation Signaling Pathway | 2.89 | 0.0469 | 2.496 | CXCL8,HMOX1,ICAM1,IRAK2,MMP3,MMP9,NFATC1,NFATC4,NFKB2,SOD2,TGFB1,TGFB3,TLR2 |
| Pancreatic Adenocarcinoma Signaling | 3.22 | 0.0748 | 2.449 | HBEGF,HMOX1,MMP9,NFKB2,PGF,TGFB1,TGFB3,VEGFA |
| STAT3 Pathway | 3.21 | 0.0672 | 2.449 | FGFR1,HGF,IL18R1,IL1RL1,IL7R,PDGFRA,PDGFRB,TGFB1,VEGFA |
| HMGB1 Signaling | 1.73 | 0.0458 | 2.449 | CXCL8,ICAM1,MAP2K3,NFKB2,SERPINE1,TGFB1,TGFB3 |
| IL-6 Signaling | 1.68 | 0.0492 | 2.449 | CXCL8,IL1RL1,MAP2K3,NFKB2,TNFAIP6,VEGFA |
| Osteoarthritis Pathway | 11.2 | 0.111 | 2.4 | ADAMTS4,BMP2,CXCL8,DDIT4,DKK1,FGFR1,FN1,IL1RL1,ITGA5,LRP1,MMP1,MMP10,MMP3,  MMP9, NFKB2, PGF, SMAD6,SMAD7,SPHK1,TGFB1,TLR2,VEGFA |
| NF-κB Signaling | 2.44 | 0.052 | 2.333 | BMP2,CARD10,FGFR1,NFKB2,PDGFRA,PDGFRB,RELB,TLR2,TNFAIP3 |
| p38 MAPK Signaling | 1.29 | 0.0439 | 2.236 | IL1RL1,IRAK2,MAP2K3,TGFB1,TGFB3 |
| Acute Phase Response Signaling | 1.14 | 0.0364 | 2.236 | FN1,HMOX1,MAP2K3,NFKB2,SERPINE1,SOD2 |
| Phospholipase C Signaling | 0.64 | 0.0258 | 2.236 | CD79B,HMOX1,ITGA5,NFATC1,NFATC4,NFKB2 |
| Synaptogenesis Signaling Pathway | 0.254 | 0.0177 | 2.236 | EPHB2,LRP1,RASGRP2,STX1A,THBS1 |
| NRF2-mediated Oxidative Stress Response | 2.83 | 0.0549 | 2.121 | FOSL1,HMOX1,MAFF,MAFG,MAP2K3,NQO1,SOD2,SQSTM1,STIP1,TXNRD1 |
| April Mediated Signaling | 2.32 | 0.103 | 2 | NFATC1,NFATC4,NFKB2,TRAF1 |
| B Cell Activating Factor Signaling | 2.24 | 0.0976 | 2 | NFATC1,NFATC4,NFKB2,TRAF1 |
| Role of IL-17F in Allergic Inflammatory Airway Diseases | 2.24 | 0.0976 | 2 | CXCL1, CXCL5,CXCL8,NFKB2 |
| FAT10 Cancer Signaling Pathway | 2.06 | 0.087 | 2 | CXCR4,NFKB2,TGFB1,TGFB3 |
| Th17 Activation Pathway | 1.52 | 0.0597 | 2 | IRAK2,NFATC1,NFATC4,NFKB2 |
| PDGF Signaling | 1.2 | 0.0471 | 2 | PDGFRA,PDGFRB,SPHK1,SYNJ2 |
| PI3K Signaling in B Lymphocytes | 1.11 | 0.0388 | 2 | CARD10,CD79B,NFATC1,NFATC4,NFKB2 |
| Gαq Signaling | 0.565 | 0.0267 | 2 | HMOX1,NFATC1,NFATC4,NFKB2 |
| T Cell Exhaustion Signaling Pathway | 0.517 | 0.0253 | 2 | NFATC1, NFATC4,TGFB1,VEGFA |
| Cardiac Hypertrophy Signaling | 0.258 | 0.018 | 2 | MAP2K3, NFATC4,TGFB1,TGFB3 |
| Leukocyte Extravasation Signaling | 2.74 | 0.0535 | 1.897 | CXCR4, ICAM1, ITGA5, MMP1, MMP10,MMP19,MMP3,MMP9,SIPA1,TIMP1 |
| B Cell Receptor Signaling | 1.42 | 0.0393 | 1.89 | CARD10,CD79B,MAP2K3,NFATC1,NFATC4,NFKB2,SYNJ2 |
| Natural Killer Cell Signaling | 2.28 | 0.0492 | 1.667 | COL11A2,COL3A1,HSPA1A/HSPA1B,HSPA6,IL18R1,MAP2K3,NFATC1,NFATC4,NFKB2 |
| ILK Signaling | 1.92 | 0.046 | 1.633 | BMP2, FLNA,FN1,MMP9,NFKB2,PGF,SNAI1,VEGFA |
| Toll-like Receptor Signaling | 3.53 | 0.0959 | 1.342 | IL1RL1, IRAK2,MAP2K3,NFKB2,TLR2,TNFAIP3,TRAF1 |
| CD40 Signaling | 2.27 | 0.0781 | 1.342 | ICAM1,MAP2K3,NFKB2,TNFAIP3,TRAF1 |
| Th2 Pathway | 2.07 | 0.0534 | 1.342 | CCR4,CXCR4,ICAM1,IL1RL1,IL24,TGFB1,TNFRSF4 |
| Factors Promoting Cardiogenesis in Vertebrates | 1.49 | 0.0444 | 1.342 | BMP2,DKK1,LRP1,TGFB1,TGFB3,WNT9A |
| Role of NFAT in Regulation of the Immune Response | 0.742 | 0.0294 | 1.342 | CD79B,NFATC1,NFATC4,NFKB2,RCAN1 |
| Role of NFAT in Cardiac Hypertrophy | 0.567 | 0.0253 | 1.342 | MAP2K3,NFATC4,RCAN1,TGFB1,TGFB3 |
| Inhibition of Angiogenesis by TSP1 | 2.64 | 0.125 | 1 | MMP9,TGFB1,THBS1,VEGFA |
| BAG2 Signaling Pathway | 2.28 | 0.1 | 1 | BAG2,HSPA1A/HSPA1B,HSPA6,NFKB2 |
| BMP signaling pathway | 1.28 | 0.05 | 1 | BMP2,NFKB2,SMAD6,SMAD7 |
| Xenobiotic Metabolism CAR Signaling Pathway | 0.511 | 0.0252 | 1 | CHST2,CYP1A1,HS3ST2,MAP2K3 |
| Sirtuin Signaling Pathway | 0.288 | 0.0186 | 1 | CXCL8,GADD45A,NFKB2,NQO1,SOD2 |
| cAMP-mediated signaling | 0.272 | 0.0184 | 1 | CCR4,DUSP4,HRH3,PDE3A |
| TGF-β Signaling | 2.95 | 0.0769 | 0.816 | BMP2,MAP2K3,SERPINE1,SMAD6,SMAD7,TGFB1,TGFB3 |
| Role of PKR in Interferon Induction and Antiviral Response | 1.84 | 0.0536 | 0.816 | HSPA1A/HSPA1B,HSPA6,IL24,MAP2K3,NFKB2,PDGFRB |
| Protein Kinase A Signaling | 1.01 | 0.0282 | 0.707 | DUSP4,DUSP8,FLNA,NFATC1,NFATC4,NFKB2,PDE3A,PTPRE,TGFB1,TGFB3 |
| eNOS Signaling | 0.572 | 0.0268 | 0 | HSPA1A/HSPA1B,HSPA6,PGF,VEGFA |
| Wnt/β-catenin Signaling | 0.81 | 0.0311 | -0.447 | DKK1,LRP1,TGFB1,TGFB3,WNT9A |
| SAPK/JNK Signaling | 1.02 | 0.0408 | -1 | DUSP4,DUSP8,GADD45A,NFATC1 |
| LXR/RXR Activation | 0.876 | 0.036 | -1 | IL1RL1,LDLR,MMP9,NFKB2 |
| PPARα/RXRα Activation | 1.07 | 0.0347 | -1.342 | HELZ2,IL1RL1,MAP2K3,NFKB2,TGFB1,TGFB3 |
| Endocannabinoid Cancer Inhibition Pathway | 0.983 | 0.0355 | -1.342 | MAP2K3,PGF,TWIST1,TWIST2,VEGFA |
| Inhibition of Matrix Metalloproteases | 5.49 | 0.189 | -1.633 | LRP1,MMP1,MMP10,MMP19,MMP3,MMP9,TIMP1 |
| PTEN Signaling | 1.66 | 0.0488 | -1.633 | FGFR1,ITGA5,NFKB2,PDGFRA,PDGFRB,SYNJ2 |
| Apelin Cardiac Fibroblast Signaling Pathway | 3.19 | 0.174 | -2 | SERPINE1,SPHK1,TGFB1,TGFB3 |
| PPAR Signaling | 1.01 | 0.0404 | -2 | IL1RL1,NFKB2,PDGFRA,PDGFRB |

| **Appendix Table 3.** Canonical Pathways of Comparison Between ***HAE Baseline vs. Non-HAE Controls Skin Biopsy Samples*** | | | | |
| --- | --- | --- | --- | --- |
| **Ingenuity Canonical Pathways** | **-log(p-value)** | **Ratio** | **z-score** | **Molecules** |
| Cardiac Hypertrophy Signaling (Enhanced) | 3.61 | 0.0175 | 2.828 | CXCL8,CXCR1,FGF18,FGFR1,IL1B,OSM,PDE4B,  WNT9A |
| Systemic Lupus Erythematosus In B Cell Signaling Pathway | 2.63 | 0.0198 | 2.236 | CXCL8,IL1B,ISG20,OSM,TRAF1 |
| Hepatic Fibrosis Signaling Pathway | 4.4 | 0.0229 | 2.121 | COL11A2,CXCL8,FGFR1,IL1B,MMP1,SERPINE1,  SNAI1, WNT9A |
| Osteoarthritis Pathway | 6.21 | 0.0402 | 1.134 | ADAMTS4,CXCL8,FGF18,FGFR1,IL1B,MMP1,  MMP3,SMAD6 |
| Natural Killer Cell Signaling | 2.34 | 0.0219 | 0 | COL11A2,FCGR3A/FCGR3B,HLA-G,HSPA6 |
| Role of Macrophages, Fibroblasts and Endothelial Cells in Rheumatoid Arthritis | 5.97 | 0.0308 |  | ADAMTS4,CXCL8,FCGR3A/FCGR3B,IL1B,  MMP1,MMP3,OSM,TRAF1,WNT9A |
| Agranulocyte Adhesion and Diapedesis | 5.78 | 0.0446 |  | CXCL1,CXCL2,CXCL8,CXCR1,IL1B,MMP1,MMP3 |
| Airway Pathology in Chronic Obstructive Pulmonary Disease | 5.59 | 0.375 |  | CXCL2,CXCL8,MMP1 |
| Hepatic Fibrosis / Hepatic Stellate Cell Activation | 5.5 | 0.0405 |  | COL11A2,CXCL2,CXCL8,FGFR1,IL1B,MMP1,  SERPINE1 |
| Granulocyte Adhesion and Diapedesis | 4.7 | 0.0392 |  | CXCL1,CXCL2,CXCL8,IL1B,MMP1,MMP3 |
| Role of IL-17A in Arthritis | 4.31 | 0.0727 |  | CXCL1,CXCL2,CXCL8,MMP1 |
| Role of Tissue Factor in Cancer | 4.2 | 0.0435 |  | CXCL1,CXCL8,IL1B,MMP1,PLAUR |
| Atherosclerosis Signaling | 4.14 | 0.0424 |  | COL11A2,CXCL8,IL1B,MMP1,MMP3 |
| Differential Regulation of Cytokine Production in Intestinal Epithelial Cells by IL-17A and IL-17F | 4.1 | 0.13 |  | CXCL1,IL1B,LCN2 |
| Role of Osteoblasts, Osteoclasts and Chondrocytes in Rheumatoid Arthritis | 3.92 | 0.0284 |  | ADAMTS4,IL1B,MMP1,MMP3,SMAD6,WNT9A |
| Glucocorticoid Receptor Signaling | 3.82 | 0.0222 |  | CXCL2,CXCL8,HSPA6,IL1B,KRT16,MMP1,SERPINE1 |
| Bladder Cancer Signaling | 3.48 | 0.0444 |  | CXCL8,FGF18,MMP1,MMP3 |
| Role of IL-17F in Allergic Inflammatory Airway Diseases | 3.34 | 0.0732 |  | CXCL1,CXCL8,IL1B |
| Oncostatin M Signaling | 3.31 | 0.0714 |  | MMP1,MMP3,OSM |
| Role of IL-17A in Psoriasis | 3 | 0.154 |  | CXCL1,CXCL8 |
| Axonal Guidance Signaling | 2.98 | 0.0161 |  | ADAM8,ADAMTS15,ADAMTS4,ADAMTS9,MMP1,  MMP3,WNT9A |
| Aryl Hydrocarbon Receptor Signaling | 2.84 | 0.0301 |  | ALDH1A3,CYP1A1,CYP1B1,IL1B |
| TREM1 Signaling | 2.74 | 0.0455 |  | CXCL2,CXCL8,IL1B |
| Differential Regulation of Cytokine Production in Macrophages and T Helper Cells by IL-17A and IL-17F | 2.71 | 0.111 |  | CXCL1,IL1B |
| Xenobiotic Metabolism Signaling | 2.63 | 0.0197 |  | ALDH1A3,CAMK1G,CYP1A1,CYP1B1,IL1B |
| HMGB1 Signaling | 2.62 | 0.0261 |  | CXCL8,IL1B,OSM,SERPINE1 |
| Bupropion Degradation | 2.53 | 0.0909 |  | CYP1A1,CYP1B1 |
| IL-17 Signaling | 2.51 | 0.038 |  | CXCL1,CXCL8,MMP3 |
